# Extracellular Vesicles as Potential Theranostic Platforms for Skin Diseases and Aging

**DOI:** 10.3390/pharmaceutics13050760

**Published:** 2021-05-20

**Authors:** Hyosuk Kim, Jong Won Lee, Geonhee Han, Kwangmeyung Kim, Yoosoo Yang, Sun Hwa Kim

**Affiliations:** 1 Center for Theragnosis, Biomedical Research Institute, Korea Institute of Science and Technology (KIST), Seoul 02792, Korea; hyoseog7@kist.re.kr (H.K.); jl5387@kist.re.kr (J.W.L.); geonhee@kist.re.kr (G.H.); kim@kist.re.kr (K.K.); 2 KU-KIST Graduate School of Converging Science and Technology, Korea University, Seoul 02841, Korea

**Keywords:** skin disease, skin aging, extracellular vesicles, biomarker, therapy

## Abstract

Extracellular vesicles (EVs), naturally secreted by cells, act as mediators for communication between cells. They are transported to the recipient cells along with cargoes such as nucleic acids, proteins, and lipids that reflect the changes occurring within the parent cells. Thus, EVs have been recognized as potential theranostic agents for diagnosis, treatment, and prognosis. In particular, the evidence accumulated to date suggests an important role of EVs in the initiation and progression of skin aging and various skin diseases, including psoriasis, systemic lupus erythematosus, vitiligo, and chronic wounds. This review highlights recent research that investigates the role of EVs and their potential as biomarkers and therapeutic agents for skin diseases and aging.

## 1. Introduction

Skin is the largest organ in the body, consisting of three distinct layers: the epidermis, the dermis, and the hypodermis. As the first defensive barrier between the body and the environment, skin is affected by intrinsic and extrinsic factors. These endogenous and exogenous factors that cause skin diseases and aging provoke skin cells and affect the biogenesis of extracellular vesicles (EVs), known as messengers for cell-to-cell communication.

**Figure 1:**
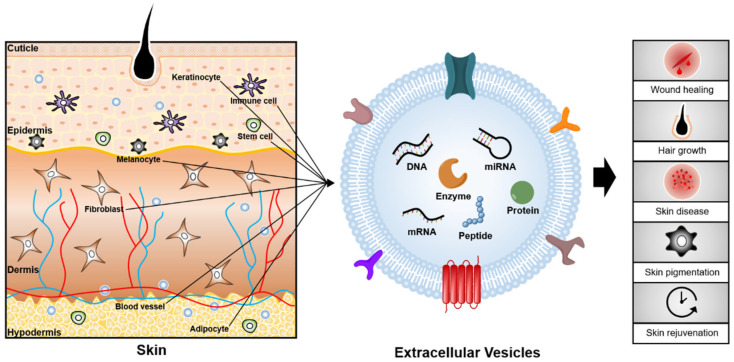
Conceptual overview of EVs and their associated effects on skin. EVs can be obtained from a variety of sources such as body fluids (blood, saliva, and serum), skin cells (keratinocytes, immune cells, fibroblasts, melanocytes, etc.), and stem cells. EVs, which carry cargoes such as nucleic acids, lipids, and proteins have the potential to be utilized as biomarkers and therapeutics for skin diseases and aging.

EVs are natural particles of a phospholipid bilayer structure that are released from almost all cells, with a diameter of 30 to 1000 nm [1]. EVs are found in various biofluids and tissues and deliver bioactive cargoes such as nucleic acids, lipids, and proteins to recipient cells [2]. Thus, EVs participate in regulating various physiological and pathological processes such as immune regulation, cell growth, and differentiation [3,4]. Recent studies indicate that EVs play a key role in skin aging and various skin diseases, including psoriasis, systemic lupus erythematosus, vitiligo, and skin wounds (Figure 1) [5,6,7,8]. These skin disorders pose a multidimensional burden that encompasses psychological, social, and financial consequences for patients, families, and society.

The differences in the level of EVs or their cargoes between patients and healthy individuals allow EVs to be used as potential biomarkers [9]. More importantly, EVs, along with their excellent biocompatibility and bioactivities, are considered ideal therapeutic agents as engineerable carriers capable of delivering various drugs. In this review, the present knowledge on the role of EVs in skin diseases and aging, EVs as therapeutics, and future challenges are discussed.

## 2. Extracellular Vesicles in Skin Diseases

Being the first line of defense, skin is constantly exposed to the external environment and, therefore, has a relatively high risk for disease. Skin disorders, which affect approximately one-third of the world population, have significant social and economic burdens [1]. Although cutaneous diseases are very prevalent, some diseases, even recently, have limited knowledge of pathophysiology. However, with the understanding of EVs as a vehicle for cell communication, better insights into the disease mechanisms can be made. This section focuses on the physiological involvement of EVs in major non-tumoral skin diseases and the promising role of EVs as diagnostic and therapeutic agents.

### 2.1. Role of Extracellular Vesicles in Skin Diseases

Psoriasis is the most common chronic inflammatory skin disorder, which affects 2–4% of the world population [10]. Psoriasis is characterized by reddish scaly papules and plaques due to abnormal proliferation of keratinocytes and increased infiltration of immune cells. Psoriasis is not a life-threatening disorder, but it can lead to psoriatic arthritis and other lethal comorbidities such as coronary artery disease, stroke, and diabetes [10]. In the case of psoriasis, the uncontrolled immune response leads to massive infiltration of the immune cells, which prompts chronic inflammation of the psoriatic lesions. The clear etiology of psoriasis has not yet been fully discovered, but a few factors have been identified as key players in the pathogenesis of psoriasis [11]. TNF-α, interleukin (IL)-23, and IL-17 are known as essential cytokines in psoriasis development [12]. The ability of EVs to carry cytokines to neighboring cells or the extracellular space of neighboring cells is pivotal in psoriasis and other inflammatory disease pathologies. As carriers for cytokines and oligonucleotides, EVs allow communication between epidermal keratinocytes and skin-infiltrating immune cells. Hence, EVs are crucial in terms of initiating and propagating the inflammation in psoriasis. Emerging evidence highlights the immunologic role of IL-17, a pro-inflammatory cytokine secreted from T helper 17 cells (T_h_ 17 cells) [13]. Levels of IL-17α in EVs of dermal dendritic cells (DCs) were correlated with the severity of the disease symptoms [14]. Additionally, microRNAs (miRNAs) contained within psoriatic keratinocyte EVs induce the polarization of T cells to a more inflamed state. Psoriatic keratinocyte-derived EVs carry miR-381 to CD 4+ T cells; the helper T cell matures to T_h_1/T_h_17, inducing further enhanced inflammatory responses [15]. Communication between innate immune cells and keratinocytes is another considerable factor that can aggravate psoriatic inflammation. As neutrophils uptake the EVs from psoriatic chemokine-induced keratinocytes, they increase the expression of pro-inflammatory cytokines [6]. Conversely, psoriatic neutrophil-derived EVs can trigger the expression of pro-inflammatory cytokines from keratinocytes, leading to ensuing immune responses [16].

Systemic lupus erythematosus (SLE) is an autoimmune disease that affects multiple organ systems. Skin is one of the main organs affected by SLE, with approximately 75% of SLE patients manifesting a skin rash or sores [17]. The cause of SLE is suspected to be multifactorial, contributed to environmental, genetic, and epigenetic factors. Autoantibody production is the main hallmark of SLE, leading to chronic inflammation and immune response directed against own cells. In SLE, circulating autoantibodies bind with nuclear proteins and form pro-inflammatory immune complexes by aggregation with target proteins. These clusters circulate and eventually deposit in tissues or interact with immune cells, which initiates cytokine production and inflammation [18]. EVs are believed to be key participants mediating both disease development and progression. High levels of immune complex (IC)-carrying EVs, which circulate in the plasma of SLE patients, are speculated as one of the possible sources of pro-inflammatory IC [19]. Upon induction, EVs isolated from SLE patients excrete more cytokines, such as IFN-α, TNF-α, IL-1β, and IL-6, all of which can serve as pro-inflammatory agents [20]. SLE patients’ EVs are filled with a higher concentration of inflammatory immunoglobulins that can damage tissues and organs [21]. Besides containing pro-inflammatory factors, EVs in SLE patients further accelerate inflammation by mediating reactive oxygen species (ROS) production and neutrophil degranulation [22]. The injurious effects of EVs in SLE patients are not limited to directly inducing inflammation. The interaction between EVs and endothelial cells prompts the secretion of chemokines and cytokines for vascular remodeling, causing a structural change in blood vessels. It can result in secondary leukocyte infiltration and exacerbation of inflammatory responses [23].

Vitiligo is an acquired skin disorder, clinically characterized by white patches due to the selective loss of melanocytes. About 0.1–2% of the world population is afflicted with vitiligo [24]. The depigmentation of the skin is from the T cell response to melanocytes, and the etiology for such self-destruction has not yet been elucidated. However, the convergence theory of integrating several genetic and environmental factors is currently accepted as the most appropriate understanding of vitiligo. Multiple pathogenesis mechanisms have been suggested, but neither of the single mechanisms can describe all the complex clinical symptoms of vitiligo. Both oxidative stress and immune system dysfunction contribute to the disease progression. Regardless of the pathogenic mechanism of the disease, the involvement of EVs in vitiligo progression is apparent. In the pathophysiology of vitiligo, EVs mediate cell communication between melanocytes and immune cells. Compared to those of a control group, vitiligo melanocytes release a higher level of the stress protein HSP 70 [25]. Ongoing clinical trials of HSP 70 as an adjuvant for vaccines further underscore its capacity to induce an immune response [26]. Unsurprisingly, the association with HSP 70 and the severity of vitiligo has been established by recent research [27]. HSP 70 from EVs interacts with antigen-presenting DC, which induces a T cell immune response towards HSP 70-secreting melanocytes. The miRNA expression profile of vitiligo lesional EVs varied from that of controls, indicating a possible role of EV-containing miRNA in disease progression. In EVs derived from vitiligo lesions, lower expression of miR-200c was found when compared to a control group [8]. miR-200c has been shown to be related to the regulation of melanin production. By suppressing the SOX1 transcription factor, miR-200c activates the β-catenin/Wnt pathway, leading to increased melanogenesis. When analyzing the miRNA contents of epidermal EVs from vitiligo patients, different miRNA profiles were observed between vitiligo lesion and non-lesion groups. Among the 29 miRNAs upregulated in the vitiligo lesional EVs, six miRNAs were chosen to transfect normal human epidermal keratinocytes in pre-miRNA form: pre-miR-202, pre-miR-185, pre-miR-525-5p, pre-miR-326, pre-miR-518a-5p, and pre-miR-518c. Following transfection with these miRNAs, all led to lower expression of melanogenesis-relevant factors, such as microphthalmia-associated transcription factor (MITF) and tyrosinase-related protein 1 and 2 [28]. Under oxidative stress, EV secretion and certain miRNAs’ expression are upregulated. According to the miRNA expression profile, a few miRNAs were upregulated in an oxidative stress model induced by hydrogen peroxide [29]. Some of these miRNAs suppressed the proliferation of melanocytes by affecting the MITF pathway and induced caspase-triggered apoptosis of the melanocytes, leading to disease progression of vitiligo.

Wound healing is a complex biological process that needs to be executed in an appropriate order and time. Wound healing consists of four phases: hemostasis, inflammation, proliferation, and remodeling [30]. During the process of wound healing, these phases need to be performed in a sequential and overlapping manner. Within 24 h, hemostasis is completed, leading to inflammation, proliferation, and remodeling; if the healing is not completed adequately, the wound may not heal and could possibly become chronic. During the process, wounded skin needs to recruit necessary cells to the site, purge the pathogens, stimulate fibroblasts, promote extracellular matrix (ECM) formation, and close the wound. EVs are speculated to accelerate the overall wound healing process, mainly by activating anti-inflammatory pathways such as the AKT/ERK and WNT pathways [31]. Nonetheless, depending on the source, EVs can serve as either pro- or anti-inflammatory factors in wound closing. From the beginning to the end, EVs from various cell types are heavily involved. Based on the source of secretion, the contents of EVs vary significantly, and each subset of EVs is involved in different stages of wound healing. Platelet-, monocyte-, and saliva-derived EVs carry tissue factors. These tissue factors activate thrombin and initiate the coagulation cascades [32]. Besides delivering tissue factors to the injured sites, EVs expedite the clotting process by serving as the surface for attachments for platelets and RBCs [7]. Hemodynamically, EVs facilitate coagulation and prevent further blood loss during the healing process. The immunomodulatory effects of EVs mainly result from intercellular communication. Both the anti-inflammatory and pro-inflammatory responses of EVs can benefit the wound healing process. During the initial phase of wound healing, removal of pathogens is required; this can be achieved through activation of the immune system. Meanwhile, in the later phase, anti-inflammatory effects can be utilized for wound healing and tissue remodeling. Depending on the source of secretion, EVs can serve as either immunosuppressants or immune-stimulants. For instance, EVs derived from monocytes behave as pro-inflammatory agents, augmenting the inflammatory response by transferring IL-1 to endothelial cells. IL-1 activates endothelial cells to increase permeability, resulting in the acceleration of immune cell infiltration in the area [33]. Contrary to the prior example, platelet-derived EVs suppress the expression of T cell-mediated cytokines [34]. Neutrophil-derived EVs can work as anti-inflammatory agents by suppressing the secretion of pro-inflammatory cytokines of macrophages and DCs. The neutrophil-derived EVs further suppress inflammation by inducing the reprogramming of immune cells to anti-inflammatory phenotypes [35].

In tissue remodeling, EVs can modulate ECM protein production. Although ECM proteins such as elastin and collagen are crucial in other phases as well, they are fundamental components of the last step of wound healing. In the maturation process, collagen III is slowly replaced with collagen I. This phase takes several days for complete restoration back to normal skin. With the treatment of EVs, the healing process and scar formation are significantly expedited [7,36]. Intestinal epithelial cell-derived EVs contained annexin A1, and these EVs triggered wound repair cascades and accelerated colonic wound closures in a mouse model. In addition, platelet-rich plasma-derived EVs induced angiogenesis and re-epithelialization in the chronic wound. However, depending on the time points of treatment, EVs may inhibit collagen expression to reduce scar formation. When adipose-derived stem cell (ADSC) EVs were treated at an early phase of the wound healing process, increases in collagen I and III were noted; when treated at a later phase, suppressed collagen production was noted [37]. In addition to affecting collagen, endothelial EVs contain matrix metalloproteinases (MMPs) and can promote angiogenesis by endothelial cell generation and migration.

### 2.2. Theranostic Applications of Extracellular Vesicles for Skin Diseases

In recent skin disease research fields, EVs derived from various cell sources have also gained much attention for theranostic translations (Table 1). Firstly, in terms of EVs as a biomarker, accurate analysis of EVs secreted from abnormal cells can provide an important disease indicator that identifies disease development and progression. Rapid and clear pathologic diagnosis is essential for the sequential treatment process. Secondly, in terms of EVs as therapeutic tools, cell-derived EVs are being proposed as new alternatives for skin diseases where the limitations of chemical, hormonal, and cellular therapies are evident. Recent studies demonstrated that stem cell-, immune cell-, and skin cell-derived EVs are involved in various stages of skin inflammation, regeneration, differentiation, and proliferation [38,39,40]. Thus, for this reason, this section will cover the overall role of EVs as biomarkers and a treatment for skin diseases.

#### 2.2.1. Extracellular Vesicles as Biomarkers for Diagnosis of Skin Diseases

The advantages of EVs as potential biomarkers for use in pathological diagnosis are derived from several unique properties. EVs contain RNAs, DNAs, proteins, and lipids, which have slightly different patterns depending on the cell type and pathophysiological conditions [41]. As the EV separation technique has progressed recently, allowing pure EVs to be extracted with a higher yield, there have been many advances in the content profiling analysis technique as well [42,43]. Through content analysis, EVs can be used as efficient disease biomarkers. EVs also have the advantage that they can be conveniently extracted from bodily fluids such as saliva, urine, and blood. For example, Byun et al. reported that there was a noticeable increase in miR-1246, miR-1290, and miR-4484 in saliva EVs of lichen planus patients [44]. It was found that miR-146a was mainly contained in urine EVs of SLE patients [45].

The EVs of SLE patients’ blood samples contained more IgG, IgM, and C1q than those of the control group [21]. Additionally, it was confirmed that miR-21 and miR-155 were significantly increased in blood EVs extracted from SLE patients [46]. Plasma EVs of patients with severe psoriasis showed significantly increased IL-17 and miR-199a [9,14]. Furthermore, Chen et al. found that five miRNAs (miR-151a, miR-199a, miR-370, miR-589, and miR-769) were increased in plasma EVs of psoriasis vulgaris patients [47]

To provide personalized treatment for each disease, an accurate diagnosis is essential. If more detailed and precise diagnosis technology of EVs is developed in the future, it will be possible to alleviate the pain of skin disease patients.

#### 2.2.2. Extracellular Vesicles as Therapeutic Tools for Skin Diseases

Stem cells with excellent differentiation and regeneration abilities are being used in therapeutics for various diseases [48,49]. In particular, stem cells have been regarded as powerful tools for tissue recovery and disease treatment due to their paracrine effect, growth promotion, and immunomodulatory properties [50]. Due to their immortality and multi-potential differentiation capacity, they are also considered therapeutic agents with infinite potential. In stem cell-based therapy, however, there are still some limitations such as high tumorigenicity, risk of immune rejection, and inappropriate differentiation [51]. To overcome these difficulties, cell-free therapy has recently received extensive interest in the regenerative medicine field. Stem cell-derived EVs (SC-EVs) are known to regulate immune balance as well as cell proliferation and regeneration by carrying miRNAs, long non-coding RNAs (lncRNAs), growth factors, and cytokines [52,53]. In particular, EVs derived from mesenchymal stem cells (MSCs) are widely used for wound healing [54,55]. Recent studies suggest that SC-EVs represent biological functions, including immunomodulation, similar to their parent stem cells [56,57]. 

In the wound inflammation phase, the expression of proper immune factors is important. miR-181c, carried by EVs of human umbilical cord mesenchymal stem cells (hUCMSCs), alleviated burn-induced inflammation through the TLR-4 signaling pathway [58]. EVs of lipopolysaccharide (LPS)-preconditioned mesenchymal stem cells modified macrophage polarization to M2 macrophages via let-7 (miRNA precursor), enhancing the anti-inflammatory effect [57]. This evidence indicates that certain miRNAs in MSC-EVs can remarkably enhance the paracrine effect of wound inflammation. The EVs of hypoxia-exposed ADSCs regulated VEGF/VEGF-R signaling, promoting angiogenesis in fat grafts [59]. miR-125a, which is abundant in ADSC-EVs, upregulated angiogenesis by inhibiting DDL4, an angiogenic inhibitor, in epithelial cells [60]. These studies suggest that ADSC-EVs promote the wound angiogenesis process. Shabbir et al. showed that MSC-EVs activated the Akt, STAT3, and ERK pathways essential for wound healing and ultimately promoted fibroblast migration and proliferation [61]. Furthermore, induced pluripotent stem cell (iPSC) EVs and ADSC-EVs containing the lncRNA MALAT1 induced the migration of fibroblasts [62,63]. Additionally, Choi et al. found that ADSC-EVs contained an abundance of miRNAs known to inhibit the expression of genes such as NPM1, PDCD4, and CCL5, promoting the proliferation and migration of skin fibroblasts [64]. ADSC-EVs could have a positive effect on wound healing by upregulating the pathways related to fibroblast migration and growth. Furthermore, it was reported that ADSC-EVs increased the expression of connective matrix and promoted wound recovery by rebalancing the ratio of collagen I and collagen III in the ECM remodeling phase [65,66].

Immune cells are involved in innate and acquired immunity and provide resistance to foreign substances invading the body to maintain immune homeostasis in the body. If the balance of immunity is compromised, chronic inflammation or autoimmune diseases may occur [74,75]. Immune cells secrete EVs by inflammatory stimulation or external stimuli, and these EVs control inflammation by mediating interactions between immune cells [76]. Accordingly, EVs derived from immune cells can be used as therapeutic agents for skin inflammatory disease therapy. Among immune cells, macrophages are essential for innate immunity and protect the body by phagocytosis against foreign pathogens and toxic substances. The activity of macrophages can be regulated through interactions with other adjacent cells, whereas activated macrophages can affect surrounding cells. Macrophages differentiate into pro-inflammatory M1 and anti-inflammatory M2 macrophages according to the given environment [77]. Recent studies found that M2 macrophage-derived EVs promoted cutaneous wound healing by reprogramming M1 macrophages into M2 macrophages [67]. Additionally, in a diabetic rat model, macrophage-derived EVs promoted wound healing by reducing pro-inflammatory factors such as TNF-α and IL-6 [68]. The phenotype of SLE may include hair loss due to destruction of the skin and blood vessel walls [78]. Wnt proteins from macrophage-derived EVs stimulated the Wnt/β-catenin signaling pathway in human dermal papilla (DP) cells [69]. The activated DP cells increased the expression of VEGF and KGF, which are essential for hair growth.

Skin cells make up the integumentary system, and keratinocytes, melanocytes, and fibroblasts are representative cell types [79]. EVs produced by skin cells have a distinct effect on skin disorders because they interact directly with other skin cells. Keratinocytes are the predominant cell type of the epidermis and protect the skin from the outside. Protein profiling studies of keratinocyte-derived EVs showed that EVs contain 14-3-3σ proteins, which are required for keratinocyte migration, and other ECM-regulating components [70]. Therefore, these EVs will be able to participate in the promotion of ECM remodeling. Melanocytes also protect skin from UV rays through melanin pigments. However, pigment deficiency causes problems such as congenital albinism and vitiligo. UV-irradiated keratinocyte-derived EVs may help in melanin deficiency disorders. Cicero et al. found that these EVs increased the expression of melanosomal proteins in melanocytes, thereby contributing to the enhancement of melanogenesis [71]. Fibroblasts are important cells for wound healing by synthesizing collagen and ECM. Fibroblast-derived EVs accelerate epidermal wound healing by reducing the expression of collagen-related miRNAs in fibroblasts and direct delivery of miR-23a [72,73]. Additionally, when these EVs are delivered to photodamaged fibroblasts, they promote the expression of glutathione peroxidase 1 (GPX-1) and collagen Ι and conversely decrease the expression of MMP 1, thus increasing the antioxidant effect of the cells [80].

Various cell-derived EVs have a promising future as biomarkers and therapeutics for skin diseases. In addition, EVs can be an alternative breakthrough for stem cell therapy in that they can overcome some of the shortcomings of cell therapy. Since the contents of EVs change depending on the cell of origin, it is important to select appropriate EVs for various skin diseases through further study.

## 3. Extracellular Vesicles in Skin Aging

Skin aging is a process in which structural integrity is decreased and normal physiological functions are disrupted by intrinsic and extrinsic factors. This section will focus on the role of EVs in skin aging and their therapeutic and cosmetic applications.

### 3.1. Role of Extracellular Vesicles in Skin Aging

Skin, an effective physical barrier between the body and the environment, ages due to intrinsic and extrinsic elements. Intrinsic aging occurs as a natural result of physiological changes over time at a genetically defined rate that cannot be altered. During this unstoppable process, skin becomes thin, dry and wrinkled and gradually undergoes atrophy. More precisely, intrinsic aging makes the epidermis about 10–50% thinner, flattens the dermo–epidermal junction, atrophies the dermis with disorganization of collagen and elastic fibers, and causes loss of adipose tissue [81]. This thinning of the epidermis and reduction in skin regeneration capacity are mainly caused by a decrease in the generation capacity of progenitor cells in the stem cell compartment that maintains physiological renewal of the epidermis and wound healing [82].

**Figure 2:**
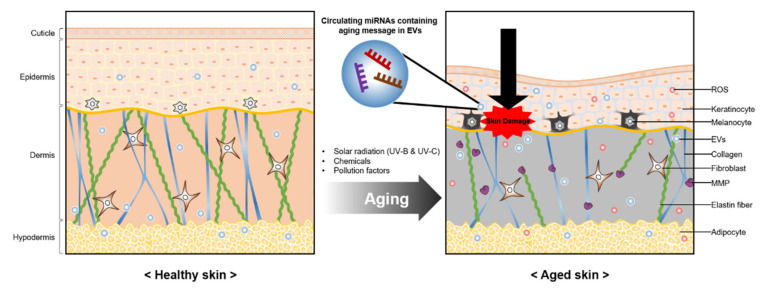
Topical skin aging caused by external factors. Skin cells that have been continuously damaged by external factors secrete miRNAs containing aging messages through EVs. These miRNAs increase ROS, melanogenesis, and expression of the MMP enzyme and conversely decrease the elastin fiber, collagen, and thickness of the epidermis.

Skin is also aged by extrinsic factors such as solar radiation, chemicals, climatic variations, and pollution (Figure 2). Among them, medium-wavelength ultraviolet radiation (UVB) and long-wavelength ultraviolet radiation (UVA) are the main players in extrinsic aging. UVB rays are limited to the superficial epidermal part of skin and cause direct damage, leading to cell senescence, apoptosis, or carcinogenesis [83]. UVA rays penetrate deeper into the dermis and generate ROS, which trigger biological changes in DNA, RNA, and proteins. ROS also activate intracellular kinases such as c-Jun N-terminal kinase (JNK), mitogen-activated protein kinase (MAPK), and extracellular regulated protein kinases (ERK), ultimately inducing transcription factor complexes of activator protein 1 (AP-1). Thus, ROS promote the expression of MMP, which increases collagen degradation and aberrant elastin accumulation [84].

In recent years, numerous studies have highlighted the key roles of lncRNAs, circular RNAs (circRNA), and miRNAs in various epigenetic changes associated with skin aging [85]. In particular, circulating miRNAs are protected from RNase degradation by packaging into EVs and transported to surrounding cells to modulate their behavior [86,87]. The circulating miRNAs are likely to be deeply linked to aging and age-related diseases. A recent study reported that senescent dermal fibroblasts, which are known to accumulate gradually in aging tissues, release more exosomes than proliferating cells do [88]. In senescent cells, exosomes and exosomal miRNAs are known to be part of the senescence-associated secretory phenotype (SASP) [88]. Indeed, one study showed that exosomal miRNAs significantly contribute to the aging process by promoting cellular senescence by inhibiting pro-apoptotic pathways [89]. In addition, circulating miR-130b was increased in obese patients, a metabolic disorder that shortens the lifespan and accelerates aging through the accumulation of advanced glycation end-products [90,91]. miR-130b inhibits the master epidermis transcription factor ∆Np63, which controls the longevity and maintenance of skin stem cells [92,93]. Interestingly, ∆Np63 negatively regulates the miR-181 family, which increases with age. The expression of miR-130b does not change in aged skin biopsy, even if the expression of miR-130b increases due to aging of keratinocytes, suggesting that the negative modulation of ∆Np63 with aging may be caused by exosomal miR-130b [93,94]. Therefore, local release of high concentrations of exosomal miR-130b from damaged or aged skin may inhibit ∆Np63 expression, thereby increasing the expression of the miR-181 cluster gene in the epidermis.

Interestingly, several studies have shown that although the expression of miR-30b, miR-181a, and miR-200c in the serum of the elderly was reduced compared to that in younger people [95,96], these miRNAs were upregulated in aged primary keratinocytes [94]. These results demonstrate that miRNAs released from cells through EVs do not necessarily reflect changes in parental cells. Little is known about the effects of EVs on skin aging. In the context of skin, the study of the role of various skin cell-derived EVs in skin homeostasis and how they can influence the genetic regulatory networks of surrounding cells is an interesting topic.

### 3.2. Theranostic/Cosmetic Applications of Extracellular Vesicles for Skin Aging

In several reports, stem cell therapy has been proven to accelerate skin repair and the wound healing process. Out of all stem cell agents, ADSCs are the most commonly used stem cell therapeutics, and their use in tissue regeneration and skin rejuvenation has been extensively studied [97,98]. Although ADSCs’ ability to multi-differentiate contributes to their therapeutic efficacy, paracrine effects of ADSCs are noted as the key mechanisms in tissue repair by modulating the local microenvironment. The cytokines that ADSCs secrete are released mainly in the form of EVs. These cytokines and growth factors act as chemoattractants, angiogenesis promoters, and pro-survival signals, all of which are crucial in skin repair [99]. Taking advantage of their paracrine effects, ADSC-free derivatives have gained attention as novel therapeutics in tissue regeneration [100]. As the ADSC derivatives are shown to improve overall skin health, the potential application of ADSC derivatives in skincare has been heavily researched [101]. Although several stem cell products are currently undergoing clinical trials, there is only one type of stem cell therapy agent approved by the FDA for clinical use, possibly because of safety concerns [102]. Without safety issues of cell therapy, ADSC derivatives, including ADSC-EVs and ADSC-CM, are already accessible to the public as cosmetic agents for skin whitening, rejuvenation, and scar prevention [103,104].

The study of ADSC-EVs is still quite limited. Most studies on ADSC derivatives in skin rejuvenation focus on ADSC-CM. Although in proteomic analysis, ADSC-CM and ADSC-EVs show slightly different protein profiles, both ADSC-CM and ADSC-EVs contained factors related to ECM organization and immunoregulation, which are crucial for skin health [105]. Whether some agents are superior in healing efficacy is not yet settled, with contrasting results shown for different medical conditions and models. In LPS-induced macrophages, ADSC-CM and ADSC-EVs were compared in terms of their anti-inflammatory effects, and interestingly, EV treatment did not show significant anti-inflammatory effects. Phagocytosis index was increased in the ADSC-EV-treated group, while ADSC-CM-treated macrophages showed a suppressed immune response [106]. In an osteoarthritis (OA) model, ADSC-CM showed a higher reduction in MMP activity and pro-inflammatory cytokine expression, while miRNA-mediated immune suppression was expected in ADSC-EV-treated groups [107,108]. In many cases, EVs have been acknowledged for their reparative efficacy: ADSC-EV treatment protected renal tissue in a hypertension model and prevented muscle damage and inflammation in a hind limb ischemic model [109,110]. Moreover, the removal of EVs from ADSC-CM negatively affected the therapeutic benefits in cell proliferation, migration, and scar prevention [111,112].

The proliferation of HDFs and HDPs is directly linked to wound healing, anti-wrinkle, aging, and overall skin homeostasis, and EVs promote skin rejuvenation by increasing the proliferation of HDFs and the synthesis of collagen and elastin in HDFs [113]. ADSC-EVs reduce the overexpression of MMPs, which degrade ECM proteins and potentially lead to wrinkles and premature skin aging [114,115].

EVs from other cellular sources are also effective in ameliorating photoaging of the skin. Bone marrow (BM)-MSC EVs reduced the photoaging in mouse skin by mediating cytokines’ expressions, lowering TNF-α and IL-1β and increasing TGF-β and CTLA 4 [116]. By reducing the senescence-associated proteins and restoring collagen, human iPSC EVs mitigated the changes that result from UVB-induced photoaging of HDFs [117]. In a nude mouse model for photoaging, 3D spheroid HDF-derived EVs decreased the signs of aging by lowering MMP expression and boosting collagen production [116].

ADSC-EVs increased the expression of essential factors for skin health (ceramides, dihydroceramide, sphingosine, and sphingosine-1-phosphate) and further promoted skin health through multiple mechanisms. Ceramides and sphingosine-1-phosphate (S1P) were found to be negatively correlated with melanin production of melanocytes, implicating the potential use of ADSC-EVs in skin-brightening agents [118]. The skin-brightening efficacy of ADSC-EVs has been evaluated in vitro and in a mouse model, and both showed reduced production of melanin [119]. However, in a clinical study of split-faced ADSC-EVs’ topical application, the brightening effect was limited and the difference in skin brightness between the EV and control groups reduced with time. Limited transdermal delivery of ADSC-EVs is often pointed out as the prominent problem of using EVs in skincare.

## 4. Topical Delivery Systems for Extracellular Vesicles

Skin permeation of EVs is often regarded as the main hurdle for EV administration. EVs show slow and limited absorption into the stratum corneum; EVs were found to reach the outermost layer by 3 h and were absorbed for 18 h after the topical administration [120]. Topically applied EVs are confined mainly in the outermost layer of the skin, the stratum corneum. Less than 1% of EVs were found to exit the stratum corneum and reach the living cells of the epidermis [121]. To overcome these barriers, several approaches, used for liposomes with poor tissue penetration properties, were devised to improve the transdermal delivery of EVs [122,123,124,125]. Different attempts at increasing EVs’ absorption were made, including the use of microneedles, needle-free injectors, and iontophoresis (Table 2). Using sponge spicules (similar to microneedles), skin absorption increased significantly and resulted in more pronounced anti-photoaging effects in a guinea pig model [126]. A keratin-based microneedle patch was integrated with EVs for subcutaneous injection, and better skin penetration with a reduced dosage was noted [127]. A needle-free injector was used for bypassing the epidermis and delivering EVs to the dermis layer [116].

In addition to the poor skin penetration of EVs, the half-life of EVs at the target site is very short due to their rapid clearance by fluids such as sweat and exposure to external factors. Thus, a sustained delivery system for EVs can be considered a key factor in order to reach the therapeutic dose of EVs in the desired site. Hydrogels have been widely used for the sustained release of drugs. They are a network of crosslinked three-dimensional hydrophilic polymers forming a matrix with a high water content [128]. Polymers commonly used to make hydrogels include natural materials such as collagen, gelatin, and chitosan; synthetic materials such as poly (ethylene glycol) (PEG) and poly (lactic acid-co-glycolic acid) (PLGA); and the combination of both. There are few reports of encapsulating EVs in hydrogels, and the field of study is still in its infancy. Wound healing was evaluated in a diabetic rat skin defect model by loading EVs derived from gingival MSCs into a chitosan/silk hydrogel sponge [129]. This non-invasive application method of EVs derived from MSCs promoted re-epithelialization and induced ECM deposition and angiogenesis, leading to effective skin regeneration. In another study on skin regeneration in diabetic mice with chronic skin wounds, Guo et al. observed the release of EVs for 4 days by loading platelet-derived EVs into a sodium alginate hydrogel [36]. There was also a study aiming to enhance wound healing with stimulating the proliferation and viability of HDF by combining chitosan and EVs released from synovium MSCs overexpressing miR-125 [130]. A homogeneous polysaccharide (ZWP) was isolated and purified from the rhizome of *Curcuma zedoaria* and loaded onto a chitosan/silk hydrogel sponge with platelet-derived EVs to confirm a wound healing result 20% faster than that on an untreated skin wound in diabetic rats [131]. Wang et al. proposed a more complex hydrogel system capable of controlled release of EVs based on antimicrobial polypeptides [132]. The pH-sensitive hydrogel, composed of pluronic F127, oxidative hyaluronic acid, and poly-ε-L-lysine, showed faster EV release in an acidic environment, lasted for 21 days, and significantly accelerated the healing rate of a diabetic full-thickness skin wound. The EV–hydrogel systems also induced the appearance of abundant skin appendages while reducing the scar tissue area. This suggests that the hydrogel system, when applied to skin wounds, has a synergistic effect on wound healing with the release of EVs. Although several attempts to improve skin penetration and the sustained release of EVs have been introduced in this section (Table 2), in-depth research through a convergence of various fields is still needed.

## 5. Conclusions and Future Perspective

In recent years, an enormous amount of research has been conducted as EVs have demonstrated potential as a therapeutic agent for nearly all diseases, not just skin diseases. In this review, we looked at the regulatory functions of EVs and their potential as biomarkers or therapeutic agents in skin diseases and aging. As naturally secreted therapeutic carriers, EVs have excellent biocompatibility with low immunogenicity compared to artificial nanoparticles such as liposomes. Furthermore, EVs have infinite potential as therapeutic agents depending on the cell source, since they deliver various bioactive factors that participate in physiological and pathological processes between cells. However, there are still some questions to be solved regarding the clinical application of EVs.

First, standardization is needed in the classification and characterization of EVs. The classification of EVs is constantly evolving, but one problem is the lack of unique markers for heterogeneous subclasses of EVs that overlap in size and content. Hence, the International Society for Extracellular Vesicles (ISEV) proposed the use of physical properties, biological composition, or condition descriptions to name subtypes of EVs [133]. Separation methods of EVs should also be standardized. A comprehensive study of the specific biological properties of each subpopulation is required through protein and nucleic acid profiling by standardization of the separation methods for isolating specific subpopulations of EVs.

Another important issue in EV studies is the low yield of EVs, which are primarily obtained in limited fluids such as culture media. Conventional methods of EV separation, such as ultracentrifugation, which require multiple steps, cause a lot of loss and damage to the EVs, which degrades the quality and purity. A study reported an increase in the yield of EVs by about 5 to 10 times when using a bioreactor [134]. Another strategy has been proposed to increase the biogenesis of EVs through overexpression of regulatory proteins involved in EVs’ biogenesis [135].

The studies on EVs as therapeutics in dermatology are just beginning, and the precise content of EVs and their multiple functions also remain to be identified. Moreover, there are some issues that should be solved in order for exosomes to be used as biomarkers for precise diagnosis. There is a need to develop a technology capable of separating exosomes with high yield and purity from a fluid sample, as well as a technology for high-sensitivity detection of key molecules. In addition, the establishment of endogenous controls for normalization of exosomal miRNAs is necessary, and large-scale studies for accurate comparison and validation of data should be conducted. Another recently discovered important function of EVs is that they are mediators of genetic exchanges between cells and are responsible for epigenetic regulation through miRNAs [87]. Moreover, it has been reported that EVs derived from senescent keratinocytes also alter the EV landscape of surrounding cells [136,137]. Thus, circulating exosomal miRNAs indicate that they have a profound relationship with skin diseases and aging, suggesting the possibility to discriminate between EV subpopulations via the cargo of these miRNAs. Continuous research on EVs will support future applications of EVs in the diagnosis and treatment of skin diseases and aging.

## Figures and Tables

**Table 1:** Summary of therapeutic applications of extracellular vesicles for skin diseases.

Disease	EV Source	Effective Molecule	Therapeutic Use	Target Cell	References
Lichen planus	Saliva	miR-1246, miR-1290, and miR-4484	Biomarker	Not determined	[44]
SLE	Urine	miR-146a	Biomarker	Not determined	[45]
SLE	Blood	IgG, IgM, and C1q	Biomarker	Not determined	[21]
SLE	Blood	miR-21, miR-155	Biomarker	Not determined	[46]
Psoriasis	Blood	IL-17	Biomarker	Not determined	[14]
Psoriasis	Blood	miR-199a	Biomarker	Not determined	[9]
Psoriasis vulgaris	Blood	miR-151a, miR-199a, miR-370, miR-589, and miR-769,	Biomarker	Not determined	[47]
Wound healing	hUCMSCs	miR-181c	Alleviates burn-induced inflammation	Macrophages	[58]
Chronic wound	LPS-preconditioned MSCs	let-7	Modifies macrophage polarization to M2 macrophage	Macrophages	[57]
Wound healing	Hypoxia-exposed adipose-derived stem cells (ADSCs)	VEGF-A and VEGF-R2	Promotes angiogenesis in fat grafts	Not determined	[59]
Wound healing	ADSCs	miR-125a	Promotes angiogenesis	Epithelial cells	[60]
Wound healing	ADSCs	Not determined	Promotes fibroblast migration and proliferation	Fibroblasts	[61]
Wound healing	iPSC	Not determined	Promotes fibroblast migration	Fibroblasts	[62]
Wound healing	ADSCs	MALAT1	Promotes fibroblast migration	Fibroblasts	[63]
Wound healing	ADSCs	miR-4484, miR-619 and miR-6879	Promotes fibroblast migration and proliferation	Fibroblasts	[64]
Wound healing	ADSCs	Not determined	Accelerates wound healing	Not determined	[65]
Wound healing	ADSCs	Not determined	Promotes ECM remodeling	Fibroblasts	[66]
Wound healing	M2 macrophages	Not determined	Enhances fibroblast migration and endothelial cell tube formation	M1 macrophages	[67]
Wound healing	Macrophages	Not determined	Reduces pro-inflammatory factors	Not determined	[68]
Alopecia	Macrophages	Wnt3a and Wnt7b proteins	Promotes hair growth	DP cells	[69]
Wound healing	Keratinocytes	14-3-3 sigma proteins	Increases MMP-1 protein	Fibroblasts	[70]
Skin pigmentation	UV-irradiated keratinocytes	Not determined	Increases melanogenesis	Melanocytes	[71].
Wound healing	Fibroblasts	Not determined	Increases collagen Ι protein	Not determined	[72]
Wound healing	Fibroblasts	miR-23a	Accelerates scratch closure	Keratinocytes	[73]

**Table 2:** Summary of therapeutic applications of extracellular vesicles for topical delivery systems.

Materials	EVs Source	Advantage	Treatment Effect	Application	References
Marine sponge *Haliclona* sp. spicule	hUCMSCs	Increased skin absorption of EVs	Promotes the expression of extracellular matrix constituents	Skin rejuvenation	[126]
Keratin hydrogel-based microneedle patch	MSCs	Enhanced treatment efficiency at a reduced dosage	Activates the hair follicle stem cells	Hair growth	[127]
Needle-free jet injector	Fibroblasts	Injection of EVs without pain	Enhances the level of dermal collagen deposition	Skin rejuvenation	[116]
Chitosan/silk hydrogel sponge	MSCs	Non-invasive application method	Promotes re-epithelialization	Skin regeneration in chronic diabetic wound	[129]
Sodium alginate hydrogel	Platelet-rich plasma	Enhanced delivery efficiency	Promotes re-epithelialization	Skin regeneration in chronic diabetic wound	[36]
Chitosan wound dressings	miR-126-overexpressing synovium MSCs	Controlled release of EVs	Stimulates the proliferation of fibroblasts and human dermal microvascular endothelial cells	Wound healing	[130]
Chitosan/silk hydrogel sponge	Platelet-rich plasma	Non-invasive application method	Promotes re-epithelialization and collagen synthesis	Skin regeneration in chronic diabetic wound	[131]
Polypeptide-based FHE hydrogel	ADSCs	Long term pH-responsive bioactive EVs’ release	Promotes re-epithelialization and angiogenesis	Skin regeneration	[132]

## Data Availability

All figures and data can be obtained by contacting the corresponding author, Sun Hwa Kim.

## Abbreviations

ADSCs—adipose-derived stem cells; BM-MSC—bone marrow-derived mesenchymal cell; DC—dendritic cell; ECM—extracellular matrix; EVs—extracellular vesicles; IFN—interferon-alpha; IL—interleukin; iPSC—induced pluripotent stem cell; lncRNA—long non-coding RNA; LPS—lipopolysaccharide; miRNA—microRNA; MMP—matrix metalloproteinase; MSCs—mesenchymal stem cells; ROS—reactive oxygen species; SLE—systemic lupus erythematosus; TNF-α—tumor necrosis factor-alpha.
